# High-Dimensional Analysis of Single-Cell Flow Cytometry Data Predicts Relapse in Childhood Acute Lymphoblastic Leukaemia

**DOI:** 10.3390/cancers13010017

**Published:** 2020-12-23

**Authors:** Salvador Chulián, Álvaro Martínez-Rubio, Víctor M. Pérez-García, María Rosa, Cristina Blázquez Goñi, Juan Francisco Rodríguez Gutiérrez, Lourdes Hermosín-Ramos, Águeda Molinos Quintana, Teresa Caballero-Velázquez, Manuel Ramírez-Orellana, Ana Castillo Robleda, Juan Luis Fernández-Martínez

**Affiliations:** 1 Department of Mathematics, Universidad de Cádiz, Puerto Real, 11510 Cádiz, Spain; Salvador.chulian@uca.es (S.C.); alvaro.martinezrubio@uca.es (Á.M.-R.); maria.rosa@uca.es (M.R.); 2 Biomedical Research and Innovation Institute of Cádiz (INiBICA), Hospital Universitario Puerta del Mar, 11009 Cádiz, Spain; 3 Department of Mathematics, Mathematical Oncology Laboratory (MOLAB), Universidad de Castilla-La Mancha, 13071 Ciudad Real, Spain; 4 Instituto de Matemática Aplicada a la Ciencia y la Ingeniería (IMACI), Universidad de Castilla-La Mancha, 13071 Ciudad Real, Spain; 5 ETSI Industriales, Universidad de Castilla-La Mancha, 13071 Ciudad Real, Spain; 6 Department of Paediatric Haematology and Oncology, 11407 Hospital de Jerez Cádiz, Spain; cristina.blazquez.sspa@juntadeandalucia.es (C.B.G.); jfrancisco.rodriguez.sspa@juntadeandalucia.es (J.F.R.G.); marial.hermosin.sspa@juntadeandalucia.es (L.H.-R.); 7 Department of Haematology, Hospital Vírgen del Rocío, 41103 Sevilla, Spain; agueda.molinos.sspa@juntadeandalucia.es; 8 Department of Haematology, Hospital Vírgen del Rocío/University of Sevilla, 41103 Sevilla, Spain; tcaballero-ibis@us.es; 9 Department of Paediatric Haematology and Oncology, Hospital Infantil Universitario Niño Jesús, Instituto Investigación Sanitaria La Princesa, 28009 Madrid, Spain; manuel.ramirez@salud.madrid.org (M.R.-O.); acastillor@salud.madrid.org (A.C.R.); 10 Department of Mathematics, Group of Inverse Problems, Optimisation and Machine Learning, University of Oviedo, 33005 Oviedo, Spain; jlfm@uniovi.es

**Keywords:** Acute Lymphoblastic Leukaemia, flow cytometry data, Fisher’s Ratio, CD38, mathematical oncology, response biomarkers, personalised medicine

## Abstract

**Simple Summary:**

B-cell Acute Lymphoblastic Leukaemia is one of the most common cancers in childhood, with 20% of patients eventually relapsing. Flow cytometry is routinely used for diagnosis and follow-up, but it currently does not provide prognostic value at diagnosis. The volume and the high-dimensional character of this data makes it ideal for its exploitation by means of Artificial Intelligence methods. We collected flow cytometry data from 56 patients from two hospitals. We analysed differences in intensity of marker expression in order to predict relapse at the moment of diagnosis. We finally correlated this data with biomolecular information, constructing a classifier based on CD38 expression.

**Abstract:**

Artificial intelligence methods may help in unveiling information that is hidden in high-dimensional oncological data. Flow cytometry studies of haematological malignancies provide quantitative data with the potential to be used for the construction of response biomarkers. Many computational methods from the bioinformatics toolbox can be applied to these data, but they have not been exploited in their full potential in leukaemias, specifically for the case of childhood B-cell Acute Lymphoblastic Leukaemia. In this paper, we analysed flow cytometry data that were obtained at diagnosis from 56 paediatric B-cell Acute Lymphoblastic Leukaemia patients from two local institutions. Our aim was to assess the prognostic potential of immunophenotypical marker expression intensity. We constructed classifiers that are based on the Fisher’s Ratio to quantify differences between patients with relapsing and non-relapsing disease. We also correlated this with genetic information. The main result that arises from the data was the association between subexpression of marker CD38 and the probability of relapse.

## 1. Introduction

Acute Lymphoblastic Leukaemia (ALL) is the most common childhood cancer, accounting for 40% of all paediatric neoplasias [1]. This disease is characterised by the abnormal growth of immature lymphocytes in the bone marrow (BM). ALLs are classified as B- or T-ALL, depending on the lineage of the cells of origin of the malignancy [2]. The former comprises the majority of cases in children and it has better prognosis than the latter. The current treatment protocols yield survival rates of around 80% [3], but the prognosis of relapsing patients is substantially worse [4]. These high survival rates are a result of combined progress in therapeutical options and diagnostic methods [5]. With respect to the former, the current options consist of multi-agent chemotherapeutic regimes, which are accompanied by Central Nervous Systems (CNS) preventive therapy and immunosuppressive drugs [6]. With respect to the latter, patients are stratified according to a risk-based scheme and treated accordingly. The criteria for risk group assignment have been refined over the years and they represent a paradigmatic cooperation of fields, including cytomorphology, cytogenetics, molecular biology, and immunobiology. High risk patients are usually defined by a high leukocyte count in peripheral blood, hypodiploid karyotype, high degree of CNS infiltration, and the presence of genetic alterations, such as BCR/ABL and MLL/AF4 translocations [7]. Improving the risk assessment is fundamental for the early identification of relapsing patients.

Among the set of diagnostic techniques, flow cytometry is employed for the identification of the immunophenotype of the clone [8], which allows quantifying tumor burden at diagnosis and follow up [9]. In fact, minimal residual disease (MRD) after one month of treatment is one of the most relevant prognostic factors [10,11]. Flow cytometry measures the surface expression levels of selected proteins for individual cells. Typical diagnostic flow cytometry studies interrogate between 10^5^ and 10^6^ cells, and the flow cytometers used in clinical contexts can detect between four and 18 markers [12]. This leads to the consistent generation of large datasets with single-cell information. However, the level of marker expression is not currently employed in risk assessment. The impediment to the full exploitation of these datasets is the complexity and scale of the data, which cannot be analysed single-handedly by the cytometrist [13]. In this sense, the routine analysis that is carried out by visualizing histograms and bidimensional plots is falling behind technical progress in the field [14].

The abundance of flow cytometry data in comparison to its restricted clinical use opened the door to the application of artificial intelligence algorithms and methods, which now conform the growing field of computational flow cytometry. Machine learning algorithms have the potential to speed up, automatise, and reduce bias in conventional analyses, but also complement the work done by the human operator [15]. Recent examples in haematology include leukocyte recognition, the prediction of refractory Hodgkin lymphoma, minimal residual disease detection in Acute Myeloid Leukaemia, risk stratification in multiple myeloma, or predicting resistance in myelodisplastic syndrome [16]. In childhood B-cell ALL, machine learning has taken advantage of clinical data in order to predict either diagnosis [17] or relapse [18], with the work of Good et al., including proteomics data for the latter purpose [19]. Reiter et al. proposed a way to automatise MRD detection in follow-up [20].

Our purpose with this work is to contribute to this growing field and show the potential of artificial intelligence applications in medical context and in childhood B-ALL specifically, which lags behind other diseases in terms of contributions. We take advantage of the high dimensionality of flow cytometry data and a multicentre database of patients in order to find differences in marker expression levels between relapsing and non-relapsing patients at diagnosis. In what follows, we detail the data collection and preprocessing steps, the feature selection by means of Fisher’s linear discriminant analysis, and the development and validation of the classifier. We also correlate it with genetic information. Finally, we compare it with current knowledge on prognostic value of protein markers and discuss the future applicability of this methodology.

## 2. Materials and Methods

### 2.1. Patients

A retrospective study was designed in accordance with the Declaration of Helsinki, and the protocol was approved by the institutional review board (IRB) of the two participating local institutions (LLAMAT Project, 2018).

The inclusion criteria for the study were ALL diagnosis between February 2009 and October 2017, age less than 19 years, and availability of BM flow cytometry data. A total of 105 patients satisfied the inclusion criteria, 62 from Virgen del Rocío Hospital (HVR) and 43 from Niño Jesús Hospital (HNJ). The exclusion criteria were the unavailability of Flow Cytometry Standard (FCS) 3.0 files, patients without a minimum of 15 immunophenotypic (IPT) markers in common with others in the dataset, and insufficient follow-up for non-relapsing patients, i.e., patients without relapse, but with less than three years after no refractory values for minimal residual disease. Finally, 56 patients were retained for further analysis. We considered the data from each hospital separately: Dataset 1 included 30 non-relapsed and eight relapsed patients from HVR, while Dataset 2 included 13 non-relapsed and five relapsed patients from HNJ. Table 1 shows the patient characteristics.

### 2.2. Flow Cytometer Machines and Antibodies

Marker expression was obtained on FACSCanto II flow cytometers, in accordance with the manufacturer’s specifications for sample preparation. The final samples were stained using an eight-colour panel with six fluorochrome-conjugated antibodies.

FCS 3.0 files contained information on forward scatter (FSC) (interpreted as size), side scatter (SSC) light (interpreted as complexity), and monoclonal antibodies used routinely in diagnosis. The markers used included B-cell (CD19, CD10, CD20, CD22, CD24, IgM, CD66c, CD79a, kappa, lambda, etc.) and T-cell-related (CD7, cyCD3) IPT markers, markers that are related to the myeloid lineage (CD9, CD13, CD33, CD123), and some general ones (CD15, CD34, CD38, CD45, CD58, CD71, HLA-DR).

### 2.3. Genetic Data

Patient data were completed with clinical characteristics at diagnosis, such as sex, age, blast percentage in bone marrow, and relapse status. Genetic characteristics were also obtained. Cytogenetic analyses were performed by conventional karyotyping and FISH, with at least 20 metaphases per sample. In the case of insufficient number of mitoses, standard karyotyping was replaced by DNA index and specific chromosomal aberrations were identified through molecular studies (RT-PCR assays).

### 2.4. Preprocessing of Flow Cytometry Files

**Figure 1:**
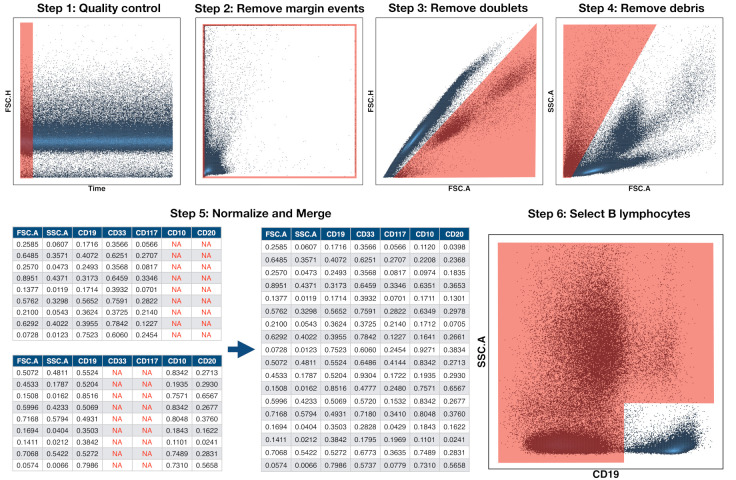
Preprocessing pipeline of Flow Cytometry Files. Preprocessing was carried out in six steps. The first four were performed in FlowJo and they consisted in the removal of abnormal acquisitions (quality control), margin events, doublets, and debris. The files were then imported into R in step 5 and, for each patient, all tubes or aliquots were merged into a single file by means of nearest-neighbour imputation. Finally, in step 6, the CD19^+^ population (B cells) was automatically selected for further analysis.

The files were first imported into FlowJo (Becton Dickinson, 10.6.1) and FACSDiva (Becton Dickinson, 8.0.1) and inspected manually. Quality control was performed and margin events, debris, dead cells, and doublets were removed, as shown in Figure 1 steps 1–4. The files were then further processed in R (3.6.0) and RStudio (1.2.1335). This software, in conjunction with Bioconductor (3.11), provides packages and methods for analysis of flow cytometry data. The tubes were compensated by means of the spillover matrix that was included in each file and then transformed with the Logicle transformation [21] included in the flowCore package (2.0.1) [22] with parameters w=0.75, t = 262,144, m=4.5, and a=0. Our next step was to bring, into a single file, the information that is contained in each of the patient’s tubes. Because each tube contains marker intensity for different markers and cells, the full set of 20 markers was not available for any of the cells, as shown in Figure 1 step 5. This posed a problem of missing data imputation, which is addressed in different ways in the context of flow cytometry [23,24,25,26]. We followed the methodology that is described in [23], which consists of nearest-neighbour imputation while using the common or backbone markers in all aliquots. The result of this process was a set of 56 files, one per patient, containing complete information on the IPT markers selected for the analysis. After this step, $10^{5}$ events were randomly sampled from each file in order to have the same number of cells for each patient.

Because data of multicentre retrospective studies can be affected by batch effects and technical variations across time and centre, we performed a normalisation that was based on a modified min-max transformation. This transformation brings all data points to the range [0,1], but it is sensitive to outliers. Instead of selecting the maximum and minimum values, we chose quantiles 0.05 and 0.95 and applied the following transformation:

(1) $$x^{\prime }=\frac{x-x_{q0.05}}{x_{q0.95}-x_{q0.05}},$$

where $x_{q0.05}$ is the 5th percentile and $x_{q0.95}$ is the 95th percentile. Finally, we used the common B-cell antigen CD19 to select the B-cell subpopulation, as shown in Figure 1 step 6. We did this in an automated way by means of the flowDensity package (1.24.0) [27], which incorporates methods for density-based cell population identification. The files were finally imported into MATLAB (Mathworks, R_2020a) via the *fca_readfcs* function [28].

### 2.5. Marker Expression Characterisation

**Figure 2:**
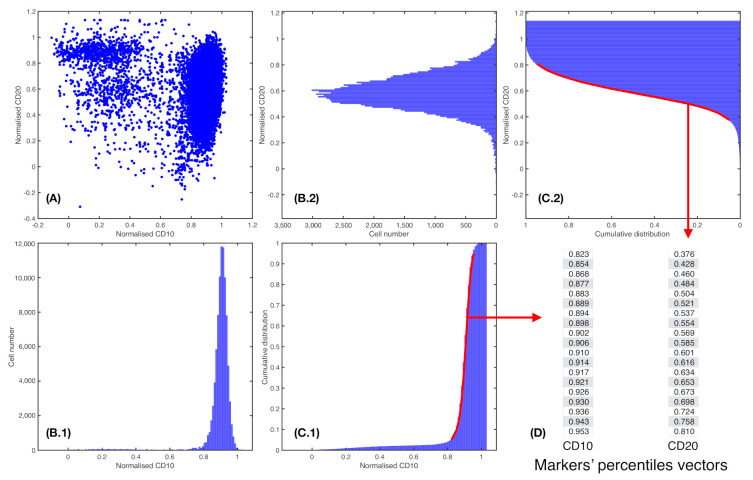
Percentile vector construction. (**A**) Scatter plot of a patient *i* for two normalised parameters, $j_{1}$ = CD10 and $j_{2}$ = CD20. (**B.1**) and (**B.2**). Histograms cell count of, respectively, $j_{1}$ = CD10 and $j_{2}$ = CD20. (**C.1**) and (**C.2**). Cumulative distribution of markers $j_{1}$ = CD10 and $j_{2}$ = CD20, respectively. In red, percentiles curve from 5th to 95th percentile. (**D**) Each percentile curve for each patient *i* and marker *j* results in a vector $x_{ij}\in R$, where *P* represents the number of percentiles chosen.

In flow cytometry, the IPT marker expression level is usually indicated by the median fluorescence intensity (MFI). In addition to this value, we also consider the mean fluorescence intensity and standard deviation. These are single-parametric representations of marker expression profiles. We fitted the cumulative distribution of every marker (Figure 2) to a generalized logistic equation in order to capture more information:

(2) $$F_{I}\left(I\right)=\frac{K}{{\left(1+Ae^{-\alpha I}\right)}^{1/\gamma }}$$

Here, *I* represents normalized marker intensity and $F_{I}$ gives the accumulated intensity. The generalized logistic equation represents families of sigmoid functions. In particular, γ=1 gives the conventional logistic equation and, as γ approaches zero, it yields the Gompertz equation. These are two well-known sigmoid functions. The carrying capacity *K* was set to 1 and initial value was fixed to the first percentile of the distribution (this determines parameter *A*). Parameter α represents the growth rate of the curve, and parameter γ provides information regarding the position of the point of maximum growth. Fitting was performed in MATLAB with function *lsqcurvefit*.

### 2.6. Fisher’s Linear Discriminant for Relapse Prediction

We consider $x_{ij}\in R^{P}$ as vectors that were obtained for each patient *i* and each common feature *j*, for i=1,...,N patients, j=1,...,M IPT markers, and *P* a number of percentiles. Thus, for each patient *i*, this results in a matrix $X\in R^{M\times P}$ of the *P* percentiles from all IPT markers *M*, as shown in Figure 2. Let us define the general Fisher’s Ratio (FR) Matrix $FR\in R^{M\times P}$ [29], where

(3) $$FR_{jk}=\frac{{\left(\mu_{R_{jk}}-\mu_{N_{jk}}\right)}^{2}}{\sigma_{R_{jk}}^{2}+\sigma_{N_{jk}}^{2}},$$

for each IPT marker *j* in each percentile *k*, for j=1,..,M and k=1,...,P. In this case, $\mu_{R_{jk}}$ and $\mu_{N_{jk}}$ are the median of percentiles *k* for the distribution of the IPT marker *j* in each class of patients. R stands for relapsing patients, while N refers to the non-relapsing ones. Parameters $\sigma_{R_{jk}}$ and $\sigma_{N_{jk}}$ are the standard deviation measures within the classes.

We can select the highest $FR_{jk}$ in order to construct a classifier, thus obtaining percentiles from several IPT markers with the lowest deviation and highest difference in median between each subset. Thus, we would obtain a general discriminant classifier of $M^{*}\leq M$ markers and associated discriminant percentiles $P^{*}\leq P$.

Let us now consider a certain IPT marker *j* and percentile *k*. We can then associate it to a specific central measure $\mu_{R_{jk}}$ or $\mu_{N_{jk}}$ and dispersion measure $\sigma_{R_{jk}}$ or $\sigma_{N_{jk}}$ for each class of patients. Thus, we set two control points

(4) $$\begin{array}{c} {\bar{R}}_{jk}=\frac{\mu_{R_{jk}}}{\sigma_{R_{jk}}},\\ {\bar{N}}_{jk}=\frac{\mu_{N_{jk}}}{\sigma_{N_{jk}}}. \end{array}$$

If we now consider a new patient that is not assigned to any set and ${\bar{v}}_{jk}$ as the value of percentiles *k* of IPT marker *j*, we can compute a control point

(5) $${\bar{P}}_{jk}=\frac{{\bar{v}}_{jk}}{\left(\frac{\sigma_{R_{jk}}+\sigma_{N_{jk}}}{2}\right)}.$$

**Figure 3:**
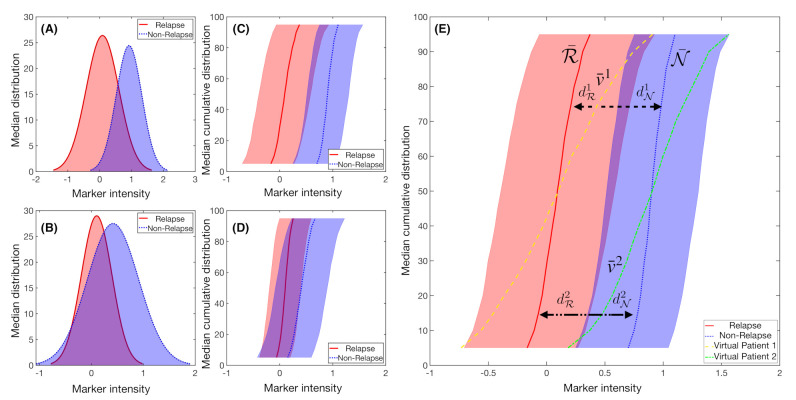
Example of synthetic IPT markers distributions. Mean distribution of a marker with, respectively, (**A**) high and (**B**) low Fisher’s Ratio, with (**C**,**D**) their respective cumulative distribution of the median ± the standard deviation values. (**E**) Median cumulative distribution of the two sets of patients for a marker with high Fisher’s Ratio. In solid red line, median cumulative distribution of relapsed patients $\bar{R}$ and in blue dotted line for the non-relapse ones. In yellow dashed line and green dashed dotted line the median cumulative distribution for the marker ${\bar{v}}^{i}$ was represented for two different virtual patients *i*. The distances to each set median, $d_{R}^{i}$ and $d_{N}^{i}$, are represented with black headed arrows, with dashed lines for Patient 1 and dashed dotted lines for Patient 2. In this example, Patient 1 would be considered to be a relapsed patient, while Patient 2 would belong in the non-relapsed set.

This point is normalised by the mean of both dispersion measures, as we consider $\bar{P}$ to be a non-assigned patient control point. Now, we can use a distance function $d:R^{k}\times R^{k}\to \left[0,\infty \right)$ to measure the separation between the new patient ${\bar{P}}_{jk}$ and the control points ${\bar{R}}_{jk}$ and ${\bar{N}}_{jk}$ (Figure 3).

We construct a probability measure P for each IPT marker and percentile as

(6) $$\begin{array}{cc} P\left({\bar{P}}_{jk}\in R\right) & =\frac{d({\bar{P}}_{jk},{\bar{R}}_{jk})}{d\left({\bar{P}}_{jk},{\bar{R}}_{jk}\right)+d\left({\bar{P}}_{jk},{\bar{N}}_{jk}\right)},\\ P\left({\bar{P}}_{jk}\in N\right) & =\frac{d({\bar{P}}_{jk},{\bar{N}}_{jk})}{d\left({\bar{P}}_{jk},{\bar{R}}_{jk}\right)+d\left({\bar{P}}_{jk},{\bar{N}}_{jk}\right)}. \end{array}$$

The mean of the probability measures for all of the IPT markers selected for each patient may allow for us to classify the patient in the relapsing or non-relapsing classes.

### 2.7. Classifier Construction and Feature Relevance

When constructing classifiers, we split the dataset in test and train set. We used the train set to select the most significant IPT markers *j* according to the Fisher Ratio ($FR_{jk}>0.5$). We used the test set in order to assess the performance of the classffier by means of a receiver operating characteristic (ROC) curve and its associated Area Under Curve (AUC). Accuracy was obtained as the number of correctly classified samples divided by the total number of classified samples. Along with these magnitudes, we computed from each confusion matrices the sensitivity, specificity, positive predictive value (PPV), and negative predictive value (NPV).

K-fold and Leave-One-Out cross-validation (LOOCV) techniques were applied. We performed each method 20 times in order to obtain a more robust measure of the performance [30] and ensure that the selection of the most important features in each classifier was not dependent on the dataset partition. For both techniques, a minimum of one patient of each set was always in the training set.

To obtain an idea of feature relevance, we performed 100 random splits with a training to test ratio of 75:25. For each split, we used the training data in order to construct a classifier and computed its performance on the test data as described above. As a result of this we gathered a collection of 100 classifiers and their performances. We next set an accuracy threshold and computed the frequency with which every marker was used in the set of classifiers that were above that threshold. This calculation was performed for different values of the accuracy threshold.

**Figure 4:**
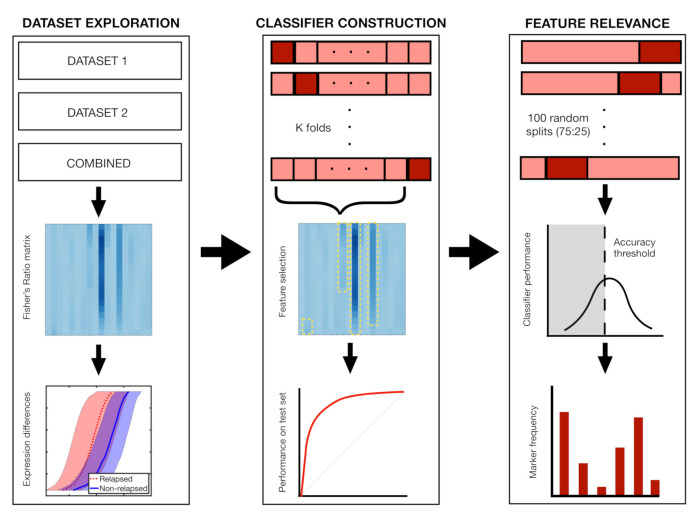
Main steps of the analysis with Fisher’s ratio. In step 1, we compute differences in marker expression between relapsing and non-relapsing patients, comparing the distributions of the most relevant markers. In step 2, we perform k-fold and Leave-One-Out cross-validation (LOOCV), constructing the classifiers with the most relevant markers of the respective train set. In step 3, we analyse the frequency with which markers are employed in 100 classifiers coming from 72:25 splits of the dataset.

Finally, we performed 100 Random Forest classifications with 50 trees each and a 75:25 split of patients in order to contrast these results with other methods. Out-of-bag error and permutation feature relevance were obtained to assess feature importance. Figure 4 shows a summary of the dataset exploration, classifier construction and feature relevance analysis.

### 2.8. Statistical Analysis

Median, mean intensity, and standard deviation were compared with *t*-tests, when considering a *p*-value that is lower than 0.05. The parameters of the generalized logistic function fit were also compared with t-tests. Correlations between percentiles of marker expression and clinical and genetic data were done with Pearson correlation coefficient. T-tests were performed to assess the significance. These computations were carried out in Python with package Pingouin [31].

## 3. Results

**Figure 5:**
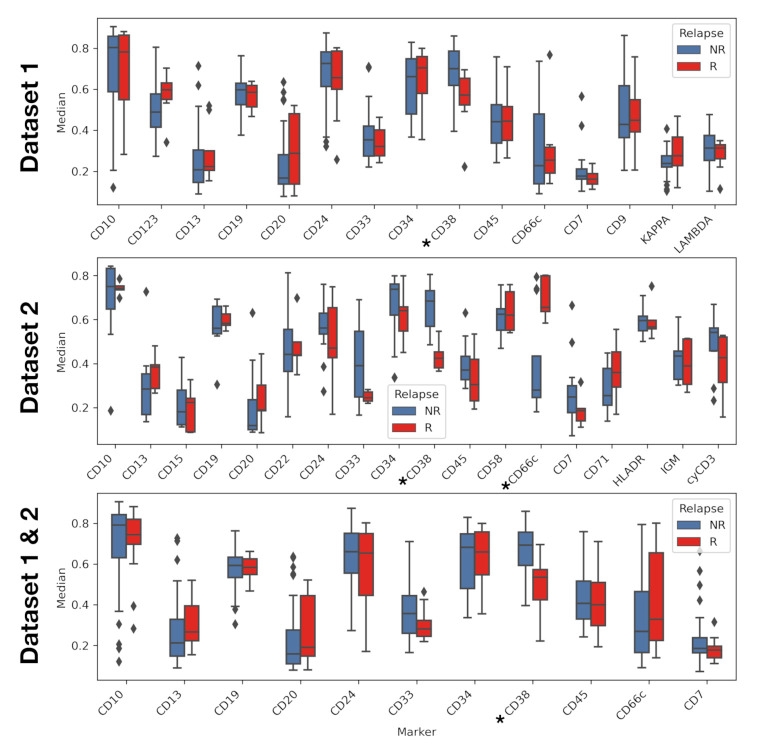
Median of immunophenotypic markers for relapse and non-relapse patients. Comparison was performed via *t*-test. Asterisk denotes markers with *p*-value lower than 0.05.

**Exploratory analysis reveals differences in marker expression and accumulated intensity profile.** Marker CD38 showed statistically significant differences in the median in all datasets, with underexpression in relapsing patients. Marker CD66c showed significant differences in median in Dataset 2, this time being overexpressed in relapsing patients (Figure 5). Mean fluorescence intensity showed the same statistical differences as the median fluorescence intensity (see Figure S1 in Supplementary Information). Statistical differences for standard deviation were only found in Dataset 2, for IPT markers CD22, and IGM. (Figure S2 in Supplementary Information)

We also fitted cumulative intensity profiles to a a generalised logistic equation (see Equation (2)). We compared the parameters α and γ between relapsing and non-relapsing patients. Statistical differences between the averages of these parameters were found for markers CD38, CD45, and CD33. However, these differences disappeared when using median parameter values (see Figure S3). Figure S4 shows an example of this fitting.

**CD38 distribution differed significantly between relapsing and non-relapsing patients.** While noting there were significant differences in MFI, we next considered the full distribution by means of the percentile curves. We employed Fisher’s ratio in order to obtain a measure of the degree of separation between relapsing and non-relapsing patients.

**Figure 6:**
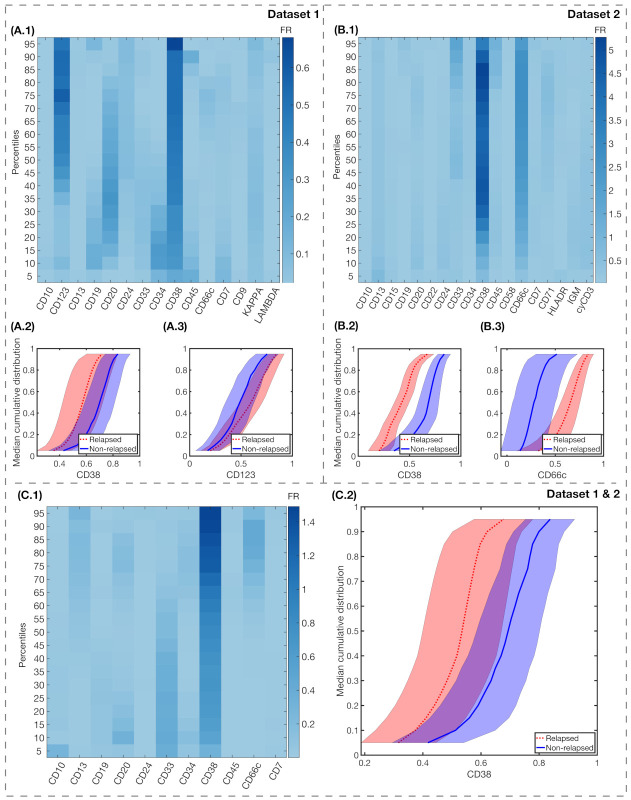
Fisher’s Ratio analyses and median cumulative distributions of markers with highest FR. Fisher’s Ratio Matrices for Dataset 1 (**A.1**), Dataset 2 (**B.1**), and both datasets combined (**C.1**). The common parameters within each dataset are represented in the x-axis, while in the y-axis we represent the percentiles of the median cumulative distribution. Colorbars show the intensity of the Fisher’s Ratio for each percentile and marker. Median cumulative distributions and standard deviation bands of the IPT markers with highest FR, for relapsed (red, dotted lines) and non-relapsed (blue, solid lines) patients are represented in the following charts: for Dataset 1, CD38 (**A.2**) and CD123 (**A.3**); for Dataset 2, CD38 (**B.2**) and CD66c (**B.3**); and, for both datasets combined, CD38 (**C.2**).

The results for FR differences between relapsing and non-relapsing patients are shown in Figure 6. We also show the median cumulative distribution of the most relevant markers. For Dataset 1, CD38 FR was high in almost all percentiles, with $FR_{jk}>0.3$, as seen in Figure 6(A.1). IPT marker CD123 had high FR for the highest percentiles, with $FR_{jk}>0.3$ for k∈(50,95). For Dataset 2, the differences between FR were significantly higher, with $FR_{jk}>3.5$ in percentiles j∈(20,95) for IPT marker CD38, and mean $FR_{jk}>2.5$ for IPT marker CD66c, as shown in Figure 6(B.1). For the combination of both datasets, only CD38 achieved a high FR with mean $FR_{jk}>0.9$ in all percentiles, as shown in Figure 6(C.1).

**Immunophenotypical markers CD38 and CD123, for Dataset 1, and markers CD38 and CD66c, for Dataset 2, predicted relapse after repeated cross-validation**. We next assessed the predictive value of the differences that were reported above, constructing classifiers, as explained in Section 2.7. We performed repeated K-fold cross validation and LOOCV. The results are shown in Table 2. We observed that the markers represented in Figure 6(A.2, A.3, B.2, B.3, C.2) were always present in the classifiers scoring high in accuracy. The maximal number of folds was determined by the number of relapsing patients (8 for Dataset 1, and 5 for Dataset 2).

**Figure 7:**
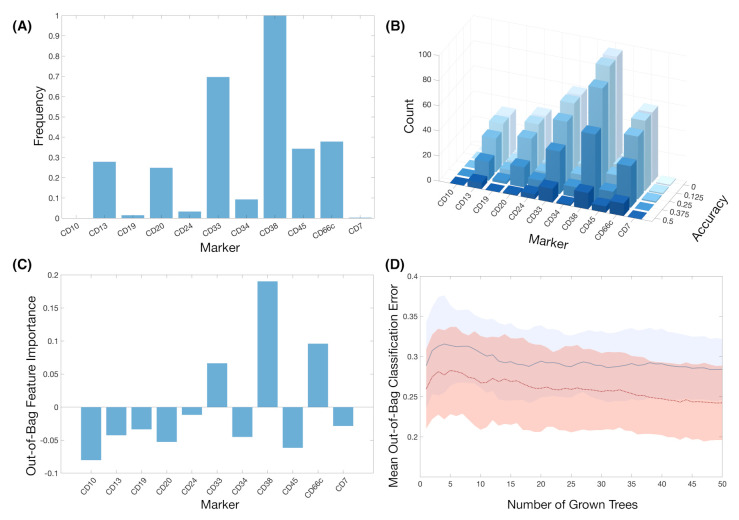
The results of feature importance analysis. (**A**) Frequency of the markers in all classifiers after 100 simulations of train-test splitting. (**B**) Histograms of the number of markers after establishing a threshold for the accuracy. (**C**) Out-of-bag feature importance of the markers after 100 Random Forests. (**D**) Mean and standard deviation bands of the Out-of-bag Classification Error in Random Forest analysis for the whole set of markers (blue, solid line) and for the set of markers with positive feature importance CD33, CD38 and CD66c (red, dotted line).

**Train-test splitting revealed other markers with potential predictive value.** We tested the predictive value of the variables by splitting all datasets (1, 2, and both combined) into training and test set with ratio 75:25. We used the training set in order to construct a classifier with the most relevant features according to FR. Figure 7A shows the frequency of the IPT markers used in the classifiers for the 100 different splits. IPT marker CD38 arose again as the marker used in all of the classifiers, while CD33 was used on almost 70% of them. We then assessed the performance of every classifier on their respective 25% test sets. Having obtained the accuracy for the 100 classifiers, we measure the frequency of IPT markers in the classifiers whose prediction accuracy was above a given threshold, as shown in Figure 7B. IPT markers CD13, CD24, CD33, CD38, CD45, and CD66c are consistently selected in classifiers with an accuracy above 50%.

**Random-Forest analysis matched the results from the constructed classifiers.** After 100 random forests, IPT markers CD33, CD38, and CD66c were the only ones with positive Out-of-bag feature importance (Figure 7C). However, after repeating the simulations only considering these markers, Out-Of Bag Classification Error was not significantly lower in comparison to the analysis with the whole set of IPT markers (mean out-of-bag error of 0.28 versus 0.31, respectively) (Figure 7D). Nevertheless, feature importance coincided with those markers with highest frequency in the previous analyses.

**Figure 8:**
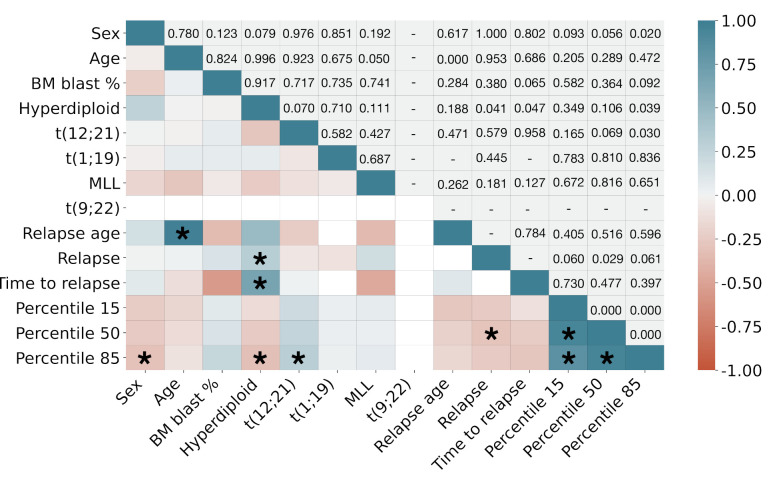
Pearson correlation coefficient between clinical, cytogenetic and marker CD38 expression data. Upper triangle shows *p*-values. Asterisks (*) denote significant correlations (p<0.05).

**CD38 marker expression correlated with genetic information.** We finally correlated marker CD38 expression with clinical and cytogenetic information. Figure 8 shows the results. For the sake of readability, we only selected percentiles 15, 50, and 85 ($P_{15},P_{50}$ and $P_{85}$) to represent low, normal, and high expression of CD38. The expected correlations include those between age and relapse age, as well as CD38 percentiles between themselves and with relapse. High CD38 expression as measured by $P_{85}$ showed significant direct correlation (p=0.03) with the presence of chromosomic alteration t(12;21) and significant inverse correlation with hyperdiplod karyotype (p=0.039). These correlations were not significant for lower percentiles. Hyperdiploid karyotype was also directly correlated with relapse and with time to relapse from diagnosis (p=0.041 and p=0.047, respectively). We also found that female patients showed a higher expression of CD38 than male patients (p=0.02).

## 4. Discussion and Conclusions

The unprecedented amount and complexity of clinical data that is available nowadays has resulted in the proliferation of bioinformatics pipelines and artificial intelligence algorithms. There are a number of issues that still hamper the integration of AI and the respective clinical context. As happens in general with the relationship between mathematics and medicine, researchers at both ends often speak a different language [32]. Many AI algorithms behave as a "black boxes", providing an outcome directly from raw data and hindering a mechanistic interpretation of the underlying phenomena. For clinical use, it is highly desirable that the features that are uncovered by these algorithms can be interpretable and actionable. As Radakovich et. al. puts it, "Algorithms can only be as clinically meaningful as the outcomes that they are designed to predict" [33].

In this work, we designed an intuitive algorithm that purports to identify patients at diagnosis with potential of relapse versus those with no risk of relapse in B-cell childhood ALL. We used flow cytometry data that were obtained at diagnosis from two local institutions and based the analysis on two concepts that are already employed in this context; the intensity and range of surface markers expression and frequency of cells within that range. Currently, this information is used for the identification and quantification of the leukaemic population, but marker expression level is not used as a prognostic factor. In our analysis, we took this into account by assigning each patient and marker its percentile curve and then used the Fisher’s ratio to look for meaningful differences between both groups of patients. That approach allowed for us to construct a classifier based on this measure in order to assess the significance of the previously obtained differences. Given the small sample size, we used the cross-validation routines to assess the validity of the Fisher’s ratio-based measure. This was later correlated with genetic information from the patients. Despite the exploratory nature of the study, we were able to find some common trends in the data.

We first carried out a more conventional analysis of marker expression by means of median and mean fluorescence intensity. This showed significant differences between relapsing of non-relapsing patients in CD38 in Dataset 1 and 2, as well as in both combined. CD66c also showed significant differences in Dataset 2. In order to look further into this, we moved from a one-parametric representation to a bi-parametric representation by means of logistic curve fitting of intensity profiles. A comparison of the fitted parameters did not yield significant results, which suggests that differences are not in the shape of the distribution but on the level of expression.

In order to delve into these differences, we compared the whole distribution by means of percentile vectors. We observed that the Fisher’s ratio displayed differences in the expression levels between relapsing and non relapsing patients. This was especially significant for the second dataset. Given that both of the datasets were pre-processed identically, the difference in the significance of the measure could be due to either sample size or different acquisition routines in either hospital. We expect to have a clearer understanding of this after increasing the number of patients in each dataset or the number of datasets as a whole. K-fold cross validation showed that, when restricting the analysis to the most important features according to the previously calculated Fisher’s ratio, the algorithm was able to better separate between relapsing and non relapsing patients, always using data only available on diagnosis.

The measurements of performance yielded good values for this biomarker, as measured by Accuracy and AUC. However, although specificity was high, we obtained a low negative predictive value, i.e., the algorithm underperformed when detecting relapses. This could be due to the intrinsic unbalance in the datasets, with only 25% of relapsed patients. The relevant information, nonetheless, was the agreement in the selection of the most important features. The Monte-Carlo based and Random Forest feature importance computation later confirmed this.

The most consistent result, in the different analyses and for both local institutions, was the association between a lower expression of CD38 marker and relapse. CD38 is a surface receptor that is present in a broad variety of immune cells. It is considered to be a cell activation marker and it operates both as a receptor and an enzyme [34]. In the B cell compartment, both bone marrow precursors and terminally differentiated cells express CD38 [35]. In the context of haematological disease, high CD38 levels have been associated with worse prognosis in Chronic Lymphocytic Leukaemia [36]. Previous studies have suggested that CD38 is a suitable therapeutic target in both AML and ALL [37,38]. There has been some controversy concerning the existence of a CD34+/CD38- population of leukaemia initiating stem cells [39,40,41,42]. In B-ALL, the accumulated evidence indicates that lower levels of CD38 could be associated with a worse outcome in terms of survival [43,44,45,46]. Our results aligned with this evidence, which suggested that a higher frequency of low CD38 expressing B cells could be an early indicator of relapse risk.

Other markers that were found to be relevant in this study were CD33 and CD66c. These two markers are normally expressed in the cells of the myeloid lineage, and they have been linked to paediatric B-ALL in the context of myeloid antigen expressing B-cell malignancies. This refers to the fact that some malignant B cells can express markers from the myeloid line. CD66c is the most frequently observed aberrant myeloid antigen in B-cell ALL. Upon studying the correlation of the expression of this antigen with known prognostic factors, previous studies have found that CD66c is associated with BCR/ABL translocation, which has been shown to confer the worst prognosis [47,48,49]. Here, we found that relapsing patients were more prone to the overexpression of this marker on diagnosis. With respect to CD33, there has been some controversy regarding its prognostic value, but evidence suggests that the presence of high expressing CD33+ cells identifies patients with worse prognosis [50], contrary to the differences that are exhibited by the percentile curves here.

The immunophenotypical marker CD123 was also highlighted by Fisher’s ratio, but only in Dataset 1. Its importance could not be further assessed since it was not available in Dataset 2. This marker was first described as a marker of Acute Myeloid Leukaemia stem cells. It was later shown to be uniformly expressed in B-ALL blasts, being proposed for the detection of minimal residual disease [51,52] and recently identified as a potential target for immunotherapies [53,54]. Interestingly, high expression of CD123 correlated with hyperdiploid karyotype, an indicator of favourable prognosis in childhood B-cell ALL [55]. In our cohort, we found a high proportion of CD123 expressing cells in relapsing patients.

Finally, we complemented the analysis of CD38 expression with clinical, cytogenetic, and molecular biology information, also relevant in the prognostic assessment of haematological diseases. CD38 intensity correlated inversely with the presence of hyperdiploid karyotypes, something that has been previously reported in the literature for the case of B-ALL [56,57]. This is interesting, because the presence of hyperdiploid karyotype is a favorable prognostic factor, while we and others found that the low expression of CD38 is an indicator of relapse. In fact, in our dataset hyperdiploid karyotype correlated with possible relapse. This is a matter of further exploration. On the contrary, the correlation between high expression of CD38 and the existence of translocation t(12;21) agrees on predicting a favourable outcome. Another interesting result is that percentile 85 presented significant correlation with a number of these features. This points out the importance of considering multi-parametric analysis of immunophenotypic markers, instead of the more conventional MFI.

In this sense, while differences in CD38 expression according to Fisher’s Ratio were present across the whole range of expression of the marker, that was not the case for CD33 and CD123. For those markers, differences were only observed in the low expression region for the former and in the high expression region for the latter. The fact that there is less evidence for their prognostic value suggests that the method presented here only leads to significant results if there is a constant difference in expression levels between both sets of patients. Indeed, this is a limitation of the study; by representing the expression as a percentile curve, we may miss information that can be clinically relevant and that refers not to the frequency of cells or intensity of expression, but to the presence or absence of a given subpopulation. In this regard, we already mentioned that a subpopulation of CD34+/CD38- cells could be associated with leukaemia initiating cells, and the same could happen for a restricted subpopulation of CD34+/CD38-/CD123+; this one agrees with the results that are presented in this paper. Another downside of using percentile curves is the high degree of correlation between percentiles of a given marker. In this sense, alternative representations should be considered when relating marker expression information to other kind of data. For example, this could be done by considering cell percentage rather than fluorescence intensity.

Another limitation of our analysis is the data, a recurrent concern in artificial intelligence in haematology [33]. Given the limited size of the dataset, conclusions should be contrasted in an extended future study. This is why we focused on building a robust methodology, keeping the exploratory nature of the work in mind. Besides, the set of relapsing patients only represented 25% of the whole dataset and unbalance could introduce biases in the analysis. A larger dataset should allow for a 50/50 analysis. Finally, there is the issue of data variability, given that it was collected retrospectively, belonging to patients from different years and hospitals. This highlights the importance of the preprocessing routine, which is also amenable to improvements in order to ensure the comparability of the samples. These weaknesses provide future lines of work. While in the process of recruiting more patients and hospitals, efforts will we directed towards the automation of the preprocessing workflow and towards the combination with more complex analyses, like dimensionality reduction, network analysis, and clustering.

Notwithstanding these limitations, this works adds to the growing field of artificial intelligence in haematology and, specifically, in B-cell childhood Acute Lymphoblastic Leukaemia. We attempted to delineate differences in marker expression between patients who relapse from the disease and those that respond to treatment, obtaining results that are directly interpretable from the clinical point of view. The main result would be the underexpression of surface marker CD38 at diagnosis in patients experiencing relapse after the first-line chemotherapy treatment. This result emerged from an unspecific comparison of intensity of marker expression and not as a particular aim of the study, which favours its extension to any other disease that uses flow cytometry as a routinary clinical tool. We hope that this contribution will be found to be useful for the purpose of exploiting flow cytometry data and marker expression level for prognostic assessment in paediatric leukaemia and other malignancies.

## Figures and Tables

**Table 1:** Patient characteristics.

	Dataset 1 (HVR) * (N = 38)	Dataset 2 (HNJ) ** (N = 18)	Total (N = 56)
Sex—no. (%)			
Male	23 (60)	9 (50)	32 (57)
Female	15 (40)	9 (50)	24 (43)
Age at diagnosis—yr/mo			
Median	3/8	3/7	3/9
Range	0/2–12/11	1/6–8/8	0/2–12/11
Long term status—no. (%)			
Relapse	8 (21)	5 (27)	13 (23)
No relapse	30 (79)	13 (73)	43 (77)
Immunophenotype—no. (%)			
Common	24 (63)	11 (61)	35 (62)
Pre-B	3 (8)	2 (11)	5 (9)
Pro-B	10 (26)	1 (5)	11 (20)
Mixed	1 (3)	2 (11)	3 (5)
BM blasts at diagnosis—%			
Median	81	90	84
Range	11–96	33–95	11–96
Karyotype—no. (%)			
Hyperdiploid (>50)	12 (32)	2 (11)	14 (25)
Normal (40–50)	15 (39)	13 (72)	28 (50)
Hypodiploid (<40)	1 (3)	0 (0)	1 (2)
Chromosomic alterations—no. (%)			
ETV6/RUNX1 t(12;21)	5 (13)	3 (23)	8 (14)
TCF3/PBX1 t(1;19)	1 (3)	1 (6)	2 (4)
MLL/AF4 t(4;11)	1 (3)	0 (0)	1 (2)
MLL rearrangement	3 (8)	0 (6)	3 (5)
BCR/ABL1 t(9;22)	0 (0)	0 (0)	0 (0)

^*^ Ten patients from Dataset 1 lacked data on karyotype; ^**^ Three patients from Dataset 2 lacked data on age, blast percentage, and cytogenetics.

**Table 2:** Validated predictive performance of best classifiers.

	Method	Accuracy	Sensitivity	Specificity	PPV	NPV	AUC
Dataset 1	LOOCV	0.75 ± 0.04	0.74 ± 0.05	0.76 ± 0.05	0.76 ± 0.04	0.75 ± 0.04	0.76 ± 0.02
	2-Fold	0.59 ± 0.1	0.63 ± 0.14	0.43 ± 0.2	0.81 ± 0.04	0.24 ± 0.12	0.56 ± 0.1
	4-Fold	0.62 ± 0.07	0.63 ± 0.1	0.58 ± 0.12	0.85 ± 0.03	0.3 ± 0.06	0.65 ± 0.06
	6-Fold	0.64 ± 0.05	0.66 ± 0.05	0.58 ± 0.13	0.85 ± 0.04	0.31 ± 0.06	0.67 ± 0.06
	8-Fold	0.7 ± 0.04	0.7 ± 0.04	0.71 ± 0.06	0.9 ± 0.02	0.39 ± 0.04	0.72 ± 0.03	
Dataset 2	LOOCV	0.66 ± 0.06	0.95 ± 0.05	0.37 ± 0.1	0.6 ± 0.04	0.88 ± 0.1	0.89 ± 0.05
	2-Fold	0.72 ± 0.07	0.95 ± 0.06	0.13 ± 0.22	0.74 ± 0.05	0.42 ± 0.41	0.68 ± 0.16
	4-Fold	0.78 ± 0.04	0.95 ± 0.05	0.34 ± 0.15	0.79 ± 0.03	0.81 ± 0.2	0.86 ± 0.06
Datasets 1 & 2	LOOCV	0.69 ± 0.05	0.62 ± 0.09	0.75 ± 0.09	0.72 ± 0.07	0.67 ± 0.05	0.78 ± 0.04
	2-Fold	0.64 ± 0.13	0.6 ± 0.17	0.75 ± 0.12	0.87 ± 0.09	0.38 ± 0.08	0.73 ± 0.11
	4-Fold	0.69 ± 0.01	0.67 ± 0.02	0.77 ± 0.01	0.91 ± 0.01	0.41 ± 0.01	0.77 ± 0.04
	6-Fold	0.7 ± 0.02	0.68 ± 0.02	0.77 ± 0.01	0.91 ± 0.01	0.42 ± 0.02	0.79 ± 0.02
	8-Fold	0.7 ± 0.01	0.68 ± 0.02	0.77 ± 0.01	0.91 ± 0.01	0.42 ± 0.02	0.79 ± 0.02
	10-Fold	0.7 ± 0.01	0.68 ± 0.02	0.77 ± 0.01	0.91 ± 0.01	0.42 ± 0.01	0.8 ± 0.02
	12-Fold	0.69 ± 0.01	0.67 ± 0.02	0.77 ± 0.01	0.91 ± 0.01	0.41 ± 0.01	0.79 ± 0.01

PPV: Positive Predictive Value. NPV: Negative Predictive value. AUC: Area under curve.

## Supplementary Materials

The following are available online at: https://www.mdpi.com/2072-6694/13/1/17/s1: Figure S1: Mean of immunophenotypic markers for relapse and non-relapse patients, Figure S2: Standard deviation of immunophenotypic markers for relapse and non-relapse patient, Figure S3: Markers with significant differences in parameters coming from intensity profiles fitting, Figure S4: Example of curve fitting of CD38 expression.

## Abbreviations

The following abbreviations are used in this manuscript:

BM	Bone Marrow
MRD	Minimal Residual Disease
FCS	Flow Cytometry Standard
IRB	Institutional Review Board
CNS	Central Nervous System
IPT	Immunophenotypic
FR	Fisher’s Ratio
CD	Cluster of Differentiation
ALL	Acute Lymphoblastic Leukaemia
ROC	Receiver Operating Characteristic
AUC	Area Under Curve
LOOCV	Leave-One-Out cross-validation
