# Supplementary material for: High-Dimensional Analysis of Single-Cell Flow Cytometry Data Predicts Relapse in Childhood Acute Lymphoblastic Leukaemia
Source: Cancers (Basel). 2020 Dec 23;13(1):17. doi: 10.3390/cancers13010017 (PMC7793064; doi:10.3390/cancers13010017)
Supplement: Supplementary file 1 [file cancers-13-00017-s001.pdf]

Article

Supplementary Materials: High-dimensional analysis of single-cell flow cytometry data predicts relapse in Childhood Acute Lymphoblastic Leukemia

Salvador Chulián, Álvaro Martínez-Rubio, Víctor M. Pérez-García, María Rosa, Cristina Blázquez Goñi, Juan Francisco Rodríguez Gutiérrez, Lourdes Hermosín-Ramos, Águeda Molinos Quintana, Teresa Caballero-Velázquez, Manuel Ramírez-Orellana, Ana Castillo Robleda, Juan Luis Fernández-Martínez

* Correspondence: victor.perezgarcia@uclm.es

Version December 2, 2020 submitted to Cancers

List of Figures

S1 Mean of immunophenotypic markers for relapse and non-relapse patients. 3
S2 Standard deviation of immunophenotypic markers for relapse and non-relapse patients. 4
S3 Markers with significant differences in parameters coming from intensity profiles fitting 5
S4 Example of curve fitting of CD38 expression 5

© 2020 by the authors. Submitted to *Cancers* for possible open access publication under the terms and conditions of the Creative Commons Attribution (CC BY) license (http://creativecommons.org/licenses/by/4.0/).

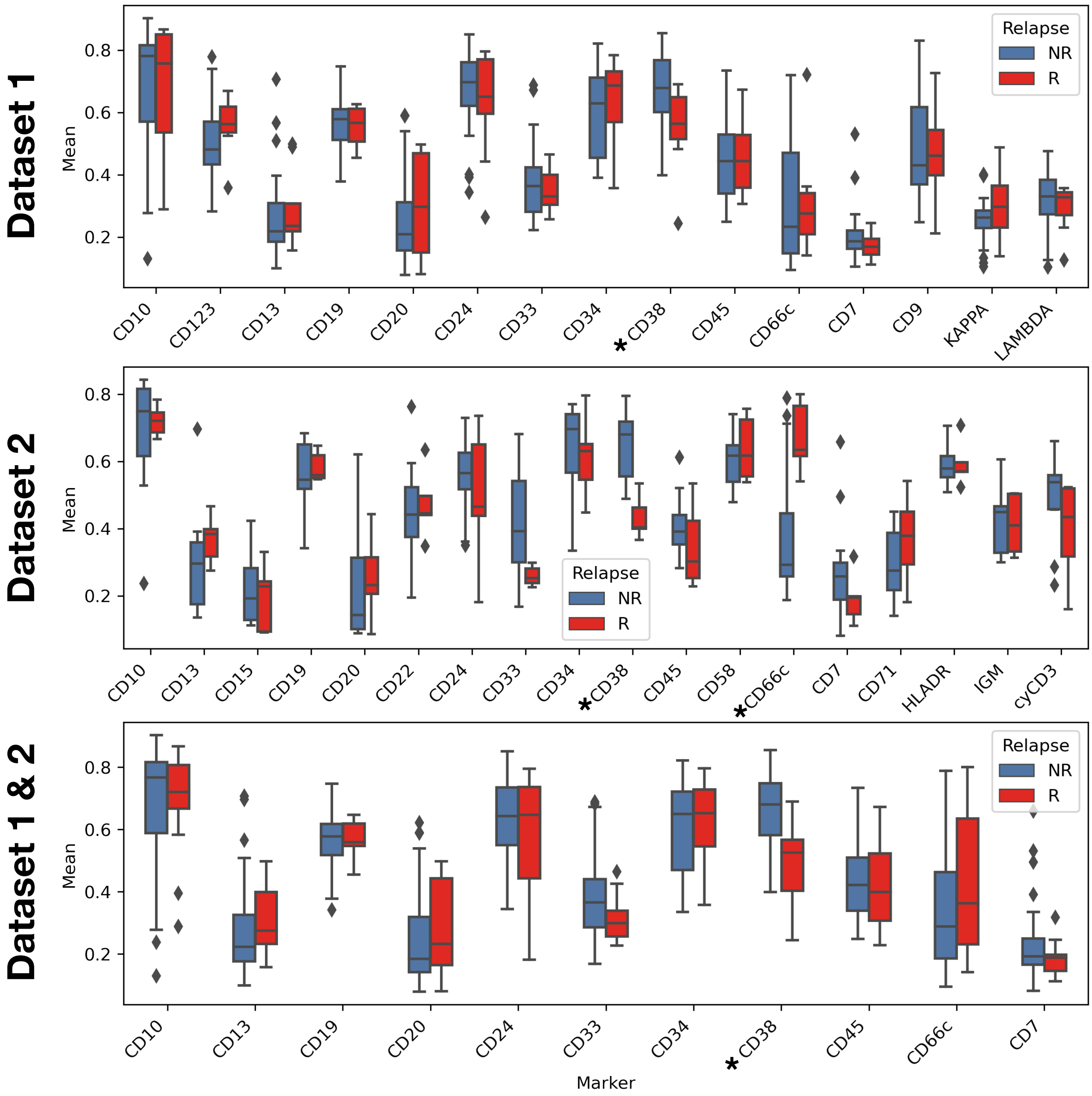

Figure S1. Mean of immunophenotypic markers for relapse and non-relapse patients. Comparison was performed via t-test and markers with differences and p-value lower than 0.05 are marked with ∗.

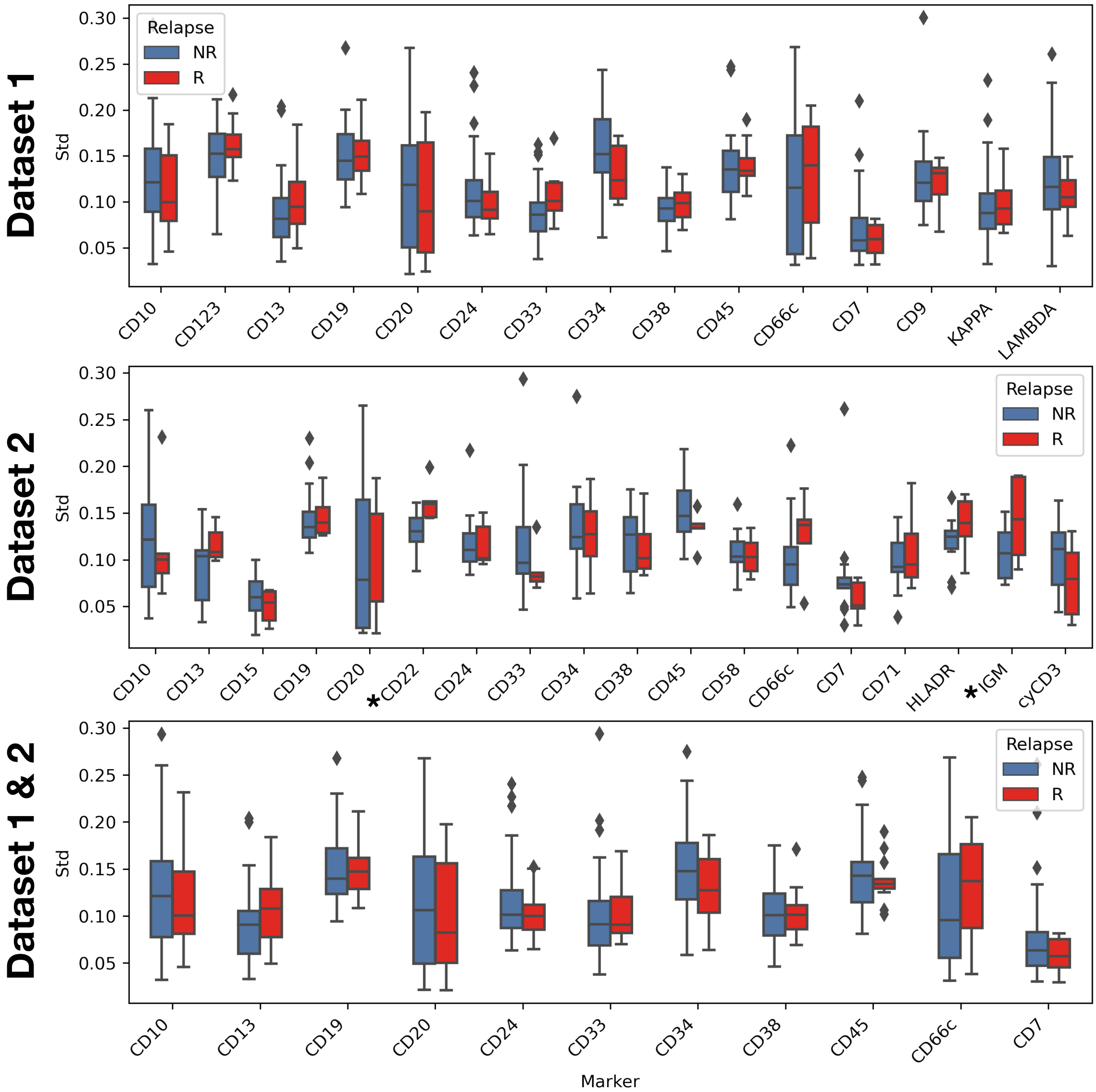

Figure S2. Standard deviation of immunophenotypic markers for relapse and non-relapse patients. Comparison was performed via t-test and markers with differences and p-value lower than 0.05 are marked with $*$.

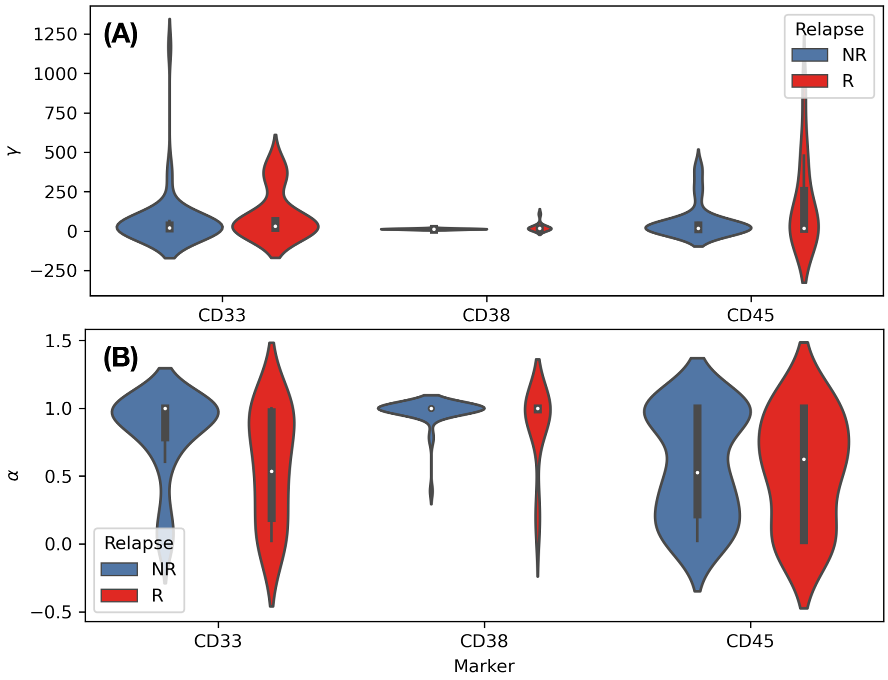

Figure S3. Markers with significant differences in parameters coming from intensity profiles fitting. **(A)** Differences for parameter γ in CD33, CD38 and CD45. **(B)** Differences for parameter α in CD33, CD38 and CD45. White dots represents median and black range represent first and third quartile.

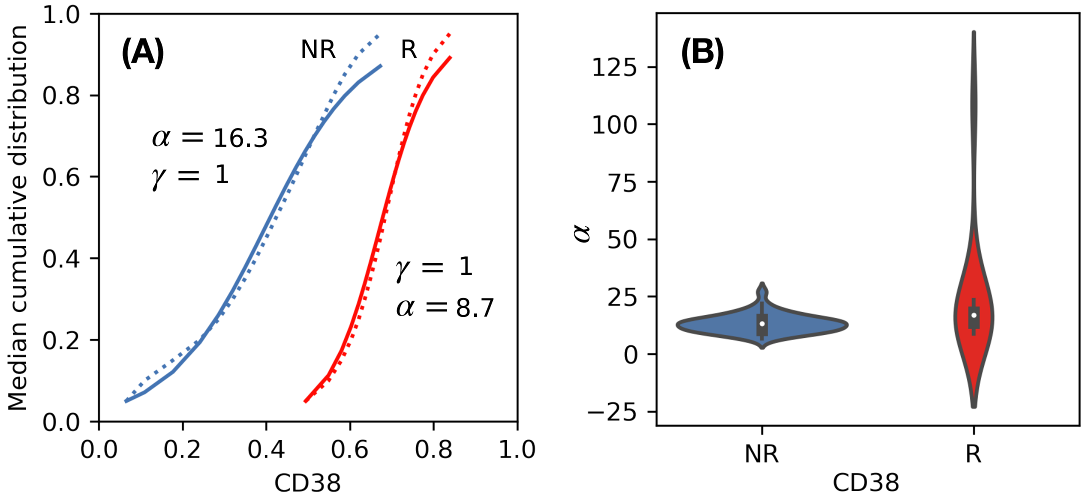

Figure S4. Example of curve fitting of CD38 expression. **(A)** Example of curve fitting for non-relapse and relapse patients. Parameters of the respective fits are included. **(B)** Differences for parameter α in CD38. We note that although having different parameter α this does not allow to distinguish between relapse and non-relapse patients.
